# Dynamics of Amino Acid Metabolism, Gene Expression, and Circulomics in a Recombinant Chinese Hamster Ovary Cell Line Adapted to Moderate and High Levels of Extracellular Lactate

**DOI:** 10.3390/genes14081576

**Published:** 2023-08-02

**Authors:** Dylan G. Chitwood, Lisa Uy, Wanfang Fu, Stephanie R. Klaubert, Sarah W. Harcum, Christopher A. Saski

**Affiliations:** 1 Department of Bioengineering, Clemson University, Clemson, SC 29634, USA; dchitwo@g.clemson.edu (D.G.C.); lisau@g.clemson.edu (L.U.); harcum@clemson.edu (S.W.H.); 2 Department of Plant and Environmental Sciences, Clemson University, Clemson, SC 29634, USA; wfu@clemson.edu; 3 Department of Chemical & Biomolecular Engineering, Clemson University, Clemson, SC 29634, USA; srklaub@g.clemson.edu

**Keywords:** lactate metabolism, eccDNA, stress adaptation, CHO, genome instability, stochastic switching

## Abstract

The accumulation of metabolic wastes in cell cultures can diminish product quality, reduce productivity, and trigger apoptosis. The limitation or removal of unintended waste products from Chinese hamster ovary (CHO) cell cultures has been attempted through multiple process and genetic engineering avenues with varied levels of success. One study demonstrated a simple method to reduce lactate and ammonia production in CHO cells with adaptation to extracellular lactate; however, the mechanism behind adaptation was not certain. To address this profound gap, this study characterizes the phenotype of a recombinant CHO K-1 cell line that was gradually adapted to moderate and high levels of extracellular lactate and examines the genomic content and role of extrachromosomal circular DNA (eccDNA) and gene expression on the adaptation process. More than 500 genes were observed on eccDNAs. Notably, more than 1000 genes were observed to be differentially expressed at different levels of lactate adaptation, while only 137 genes were found to be differentially expressed between unadapted cells and cells adapted to grow in high levels of lactate; this suggests stochastic switching as a potential stress adaptation mechanism in CHO cells. Further, these data suggest alanine biosynthesis as a potential stress-mitigation mechanism for excess lactate in CHO cells.

## 1. Introduction

In 2021, global sales of biopharmaceuticals reached an all-time high of $343 billion. A majority of biopharmaceutical sales were generated from monoclonal antibody (mAb) products, which are often commercially produced in Chinese hamster ovary (CHO) cell lines. CHO cell lines are broadly utilized due to the relative ease of the culture, non-susceptibility to viruses, and thorough history of regulatory approval. The current industry standard for CHO cell manufacturing is fed-batch cultures in which nutrients are gradually added to a culture over an extended period; however, fed-batch operation does not allow for an easy removal of metabolic waste products, such as lactate and ammonia. Limiting lactate production in CHO cell cultures has been thoroughly studied as excessive lactate in cell cultures imparts a suboptimal environment by increasing acidity in uncontrolled cultures [1,2], negatively impacts viable cell density (VCD) [3], and reduces both cell-specific and overall recombinant protein productivity [3,4]. Strategies to limit or eliminate lactate from cultures include the pH-controlled delivery of glucose [5], cell line engineering [6,7] (U.S. patent No. US11242510B2), and controlled lactate feeding [8]. The detrimental impacts of lactate can be observed at concentrations as low as 20 mM, and cultures are often unable to recover when the concentration exceeds 40 mM [9]. Often, lactate is produced in the early stages of a fed-batch culture and accumulates until a metabolic switch causes the cells to consume the excess lactate [10,11,12]. While the exact mechanisms behind this switch are unclear, multiple hypotheses have been proposed to explain this observation, such as the depletion of glucose and/or glutamine [13,14,15,16,17], shifts in pH and/or temperature [18,19], and an increased oxidative capacity [20].

In addition to metabolic waste accumulation, cells grown in a suspension culture experience other stresses such as shear stress [21] and nutrient depletion [22]. Multiple stress response mechanisms exist to mitigate these challenges at a variety of levels such as substrate [23], gene expression [24], and gene regulation [25]. Recent studies have identified a microevolutionary stress adaptation mechanism with gene overexpression via extrachromosomal circular DNAs (eccDNAs) [26,27,28,29]. EccDNAs are a common vehicle for gene amplification, which may correlate with gene overexpression in a subpopulation of cells [28,30,31]. EccDNAs are generated from the genome through multiple biogenesis pathways [32,33,34,35]. The genetic heterogeneity of cells is perpetuated by the randomness of eccDNA generation and recombination [36]. This study characterized the relationship between lactate adaptation and genetic changes within CHO cells that may mediate the adaptation via eccDNA microevolution and/or transcriptomic shifts. A recombinant CHO K-1 cell line was gradually adapted to higher levels of extracellular lactate in 10 mM increments up to 60 mM in shake flask cultures. Batch cultures were sampled daily to characterize growth profiles, amino acids, and other key metabolites. Additionally, cells were also harvested at two levels of lactate adaptation for an eccDNA and transcriptome analysis. EccDNAs were purified, sequenced, and annotated for gene content. Transcriptome data were then integrated to identify eccDNA-derived transcripts and expression profile patterns.

## 2. Materials and Methods

### 2.1. Cell Lines and Batch Culture Adaptation

**Figure 1:**
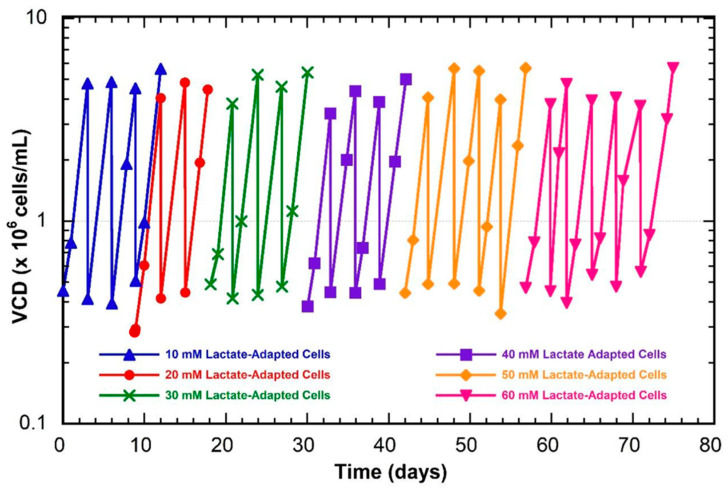
Growth profiles during the lactate-adaption process. Cells were gradually passaged into higher lactate concentrations at 10 mM increments. 10 mM adaptation (blue triangles), 20 mM adaptation (red circles), 30 mM adaptation (green crosses), 40 mM adaptation (purple squares), 50 mM adaptation (yellow diamonds), and 60 mM adaptation (pink inverted triangles).

The cell line used in this study was a recombinant CHO K-1 cell line that expresses an anti-HIV monoclonal antibody (VRC01); the cells were generated and donated by the NIH. All cultures were grown in ActiPro media (Cytiva) supplemented with 6 mM of glutamine. During the adaptation process, cells were cultured in 125 mL baffled, vented shake flasks (VWR^®^, Radnor, PA, USA) with a 30 mL working volume. Cultures were maintained in an incubator at 37 °C and 5% CO_2_ with a shake speed of 180 rpm (0.75 inch throw). Cells were progressively passaged into higher concentrations of lactate in 10 mM increments up to 60 mM. Adaptation was achieved when three criteria were met: (1) the growth rate in lactate-supplemented media was equivalent to that of unadapted cells in lactate-free media, (2) the VCD reached 4 × 10^6^ cells/mL by Day 3, and (3) the first two criteria were both sustained over three passages. After adaptation, cells were banked in a 10% dimethylsulfoxide (DMSO) media and stored in liquid nitrogen. Stocks from the liquid nitrogen were then thawed before beginning the next adaptation step (Figure 1).

Frozen stocks of unadapted, 30-mM-adapted, and 60-mM-adapted cells were rapidly thawed into media containing the respective amount of lactate. Cells were seeded into new flasks 3 days after thawing with a target seeding density of 0.5 × 10^6^ cells/mL. Triplicate cultures (*N* = 3) were used for each condition; lactate-adapted cells were also grown in a medium without supplemental lactate. Incubators were maintained at 37 °C and 5% CO_2_ with an agitation rate of 180 rpm. Flasks were sampled daily for viable cell density (VCD), viability, metabolites, and amino acids. Viability and VCD were measured on a Vi-CELL^TM^ XR Cell Viability Analyzer (Beckman Coulter, Brea, CA, USA). Glucose, lactate, glutamine, glutamate, ammonia, and IgG were measured on a Cedex Bio Analyzer (Roche Diagnostics, Basel, Switzerland). Human IgG was used for Cedex quantification. The Cedex calibrators used for standardization were Calibrator A Bio (06682189001) and Calibrator B Bio (06682553001). Concentrations of amino acids, with the exception of glutamine and glutamate, were determined via capillary electrophoresis with high pressure mass spectrometry (CE-HPMS) using a REBEL (908 Devices, Boston, MA, USA). Samples were diluted 1:100 in a REBEL diluent and analyzed with the REBEL Spent Media Analysis Kit V2 (850-00050) per the manufacturer’s recommended protocol.

### 2.2. EccDNA Enrichment and Sequence Library Preparation

Unadapted, 30-mM-adapted, and 60-mM-adapted cells were thawed from liquid nitrogen stocks into respective media. Cultures were passaged after 3 days into duplicate flasks (*N* = 2) and harvested 3 days later in early exponential growth. Cell pellets of approximately 4 × 10^6^ cells were collected from each flask, placed in RNAlater (Thermo, AM7020), and kept at −20 °C until library preparation. Approximately 1 × 10^6^ cells from each pellet were used for total RNA and genomic DNA (gDNA) extraction. Extractions were performed using an RNEasy midi kit (Qiagen, 74004) and a DNEasy Blood and Tissue kit (Qiagen, 69504) per the manufacturer’s recommended protocols. Total gDNA was quantified with a Qubit fluorometer 2.0 (Thermo, Q32866) before it was used as starting material for a Phi-29-mediated rolling circle amplification (RCA) and magnetic bead purification (KAPA Pure Beads, Roche, KK8000) as described in the CIDER-seq protocol [37]. EccDNA libraries had SMRTbell barcodes adapted to the sequences by a third-party vendor before sequencing on a PacBio Sequel II with HiFi reads. RNA samples were treated with a DNAse solution prior to sample quantification. A NEBNext Ultra II RNA Library Prep Kit for Illumina was used for library preparation per the manufacturer’s recommended procedures and pooled in equimolar ratios for sequencing. Paired-end reads for each sample (2 × 150 bp) were collected on an Illumina NovaSeq 6000 S4 flow cell to an approximate depth of 20 million read pairs.

### 2.3. Bioinformatic Pipeline

Raw eccDNA sequencing data were processed using the DeConcat algorithm from the CIDER-seq protocol [37] to confirm circularity and genomic origin. Sequences for biological replicates for each condition (*N* = 2) were combined together before clustering to a 90% sequence identity threshold using CD-Hit [38]. Raw RNA-seq data had sequencing adapters removed with Trimmomatic [39] before checking data quality with FastQC. Reads were then aligned to the reference transcriptome with Bowtie2 [40]. Transcript abundance was calculated with RSEM [41], which was used to calculate differential expression with edgeR [42].

### 2.4. EccDNA Structure and Identification of Genomic Origins

BLAST [43] was used to annotate origins of replication (ORIs) and ribosomal DNAs (rDNAs) on observed eccDNAs. Custom databases were made by collecting known mammalian origins of replication (*N* = 118, retrieved on 14 July 2022) and rDNA genes in humans (*N* = 804), mice (*N* = 81), rats (*N* = 153), and Chinese hamsters (*N* = 25) (retrieved on 9 January 2023) from NCBI. BLAST searches were conducted with an e-value of 1 × 10^−50^. Databases for custom ORI and rDNA BLAST searches are shown in Supplemental Tables S1 and S2, respectively. Transfer RNAs (tRNAs) were identified using tRNAscan-SE 2.0 [44]. Repetitive motifs on eccDNAs were annotated and masked using RepeatMasker (Smit, AFA, Hubley, R & Green, P. *RepeatMasker Open-4.0*.2013-2015 http://www.repeatmasker.org, accessed on 31 July 2022). Repeat-masked sequences had genes annotated using Maker [45]. Observed eccDNAs were BLASTed against the PICRH Chinese hamster reference genome [46] to find the most likely genomic origin of sequences. Parameters used for BLAST searches are the same as those for ORI and rDNA annotation. The highest-scoring BLAST results for each eccDNA were then assigned to 500 kbp windows of the genome created using BEDTools [47]. Counts of eccDNAs per genome window were visualized with Rideogram [48]. Z-scores were assigned to windows to identify statistically significant regions of eccDNA biogenesis.

## 3. Results

### 3.1. Phenotypic Cell Culture Data

**Figure 2:**
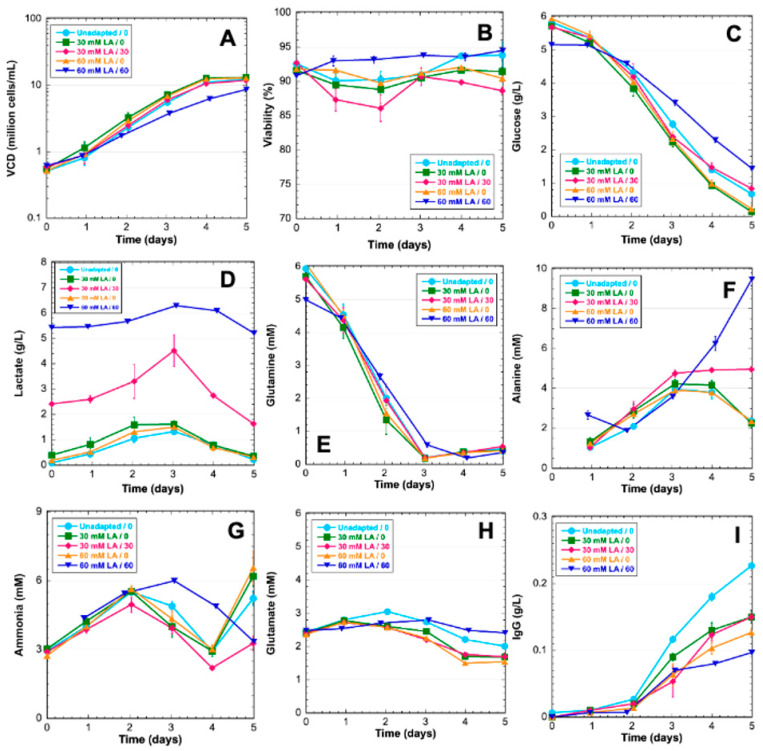
Growth characteristics for unadapted and lactate-adapted VRC01 CHO cells cultured in various levels of lactate. (**A**) Viable cell density (VCD), (**B**) Viability, (**C**) Glucose, (**D**) Lactate, (**E**) Glutamine, (**F**) Alanine, (**G**) Ammonia, (**H**) Glutamate, (**I**) IgG. LA—lactate-adapted. Unadapted cells in normal media (light blue circles), 30-mM-adapted cells in normal media (green squares), 30-mM-adapted cells in lactate-supplemented media (pink diamonds), 60-mM-adapted cells in normal media (yellow triangles), 60-mM-adapted cells in lactate-supplemented media (dark blue inverted triangles).

Unadapted, 30-mM-adapted, and 60-mM-adapted cells were grown in 5-day batch cultures to characterize the lactate-adapted phenotype. Cells were grown in media supplemented with lactate that corresponds to adaptation levels. Further, 30-mM-adapted and 60-mM-adapted cells were also grown in a medium with no supplemental lactate. Lactate adaptation had a small effect on growth rate for the 60-mM-adapted cultures with supplemental lactate as the maximum viability being 9.6 × 10^6^ cells/mL by Day 5; however, all other cultures achieved a maximum VCD of 1 × 10^7^ cells/mL (Figure 2A). Cell viability was also similar across conditions by the end of the cultures (Figure 2B). Glucose was a primary carbon source in all cultures; however, lactate-adapted cells without supplementation with lactate utilized more glucose than unadapted cells and lactate-adapted cells in media with supplemented lactate. There is no indication lactate is being utilized for gluconeogenesis as there is no observed accumulation of glucose (Figure 2C). Lactate levels in all cultures increased before cells switched to lactate consumption on Day 3 regardless of lactate adaptation or supplementation (Figure 2D). The metabolic switch to lactate consumption correlated with glutamine depletion (Figure 2E). Alanine increased in all cultures until Day 3. After Day 3, alanine declined in cultures without supplemental lactate, but plateaued in 30-mM-adapted cultures supplemented with lactate and continued increasing in 60-mM-adapted cultures supplemented with lactate (Figure 2F). The ammonia profiles pair closely in all experimental conditions until Day 3. Lactate-adapted cultures in media with supplemental lactate did not experience the late culture accumulation of ammonia as seen in all other conditions (Figure 2G). Glutamate concentration paired closely among all experimental conditions throughout the culture. The consumption of glutamate was first observed on Day 3 in line with glutamine depletion and lactate consumption (Figure 2H). Titer was observed to be significantly higher in unadapted cells. Lactate adaptation resulted in lower titer with 60-mM-adapted cultures showing lower titer than 30-mM-adapted cultures (Figure 2I).

### 3.2. EccDNA Sequence Composition

**Figure 3:**
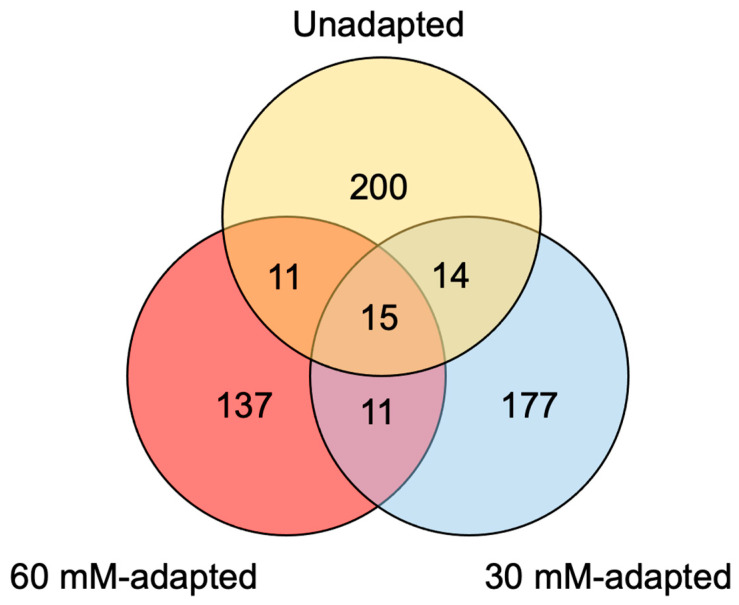
EccDNA-encoded gene distributions for CHO cells gradually adapted to higher levels of extracellular lactate. Unadapted (yellow), 30-mM-adapted (blue), and 60-mM-adapted (red).

Cell pellets for the eccDNA analysis were collected on Day 3 during the exponential growth phase. The number of observed eccDNAs decreased as the lactate adaptation level increased, though this may be due to natural variation as individual library preparations of the same sample can show a high degree of heterogeneity [49]. The average length of observed eccDNAs was similar between unadapted and 30-mM-adapted; however, the observed average eccDNA sequence length was approximately 500 bp higher for the 60-mM-adapted samples. Many sequence structures were observed to be similarly distributed for the unadapted and lactate-adapted cultures including the percentage of bases pertaining to repeats (~38.0%), GC content (~40.5%), and tRNA genes (~9.3%). Full summaries of tRNA annotation for unadapted, 30-mM-adapted, and 60-mM-adapted eccDNAs are available in Supplemental Tables S3–S5, respectively. Notably, the proportion of eccDNAs encoding genes increased to 5.2% in the 60-mM-adapted samples from 4.0% in the 30-mM-adapted samples—a clear overrepresentation of gene content relative to the genome and other eccDNA samples. Origins of replication (ORIs) and ribosomal DNAs (rDNAs) were identified in all three conditions, but sequences with a ≥95% identity to ORI or rDNA sequences were not substantial (≤6 per condition). Full BLAST output tables for the ORI analysis are available in Supplemental Tables S6–S8. Full BLAST output tables for rDNA annotation are available in Supplemental Tables S9–S11 for unadapted, 30-mM-adapted, and 60-mM-adapted eccDNAs, respectively. An analysis of repeat and retrotransposable elements revealed that short interspaced nuclear elements (SINEs) showed a gradual decrease in abundance as adaptation progressed, while long interspaced nuclear elements (LINEs) showed a gradual increase in abundance. However, these changes demonstrated less than a 1% difference between unadapted and 60-mM-adapted samples and could be within the range of normal variation. EccDNAs were analyzed for the genuine coding sequence, which identified 567 genes across the three conditions. Our analysis revealed the presence of 240 genes in unadapted samples, 217 genes in 30-mM-adapted samples, and 174 genes in 60-mM-adapted samples. Notably, a minimal overlap of eccDNA genes was observed across lactate adaptation levels, with only 53 genes found to be present in multiple conditions. Homology-based functional annotations for unadapted, 30-mM-adapted, and 60-mM-adapted eccDNAs are available in Supplemental Tables S12–S14, respectively. Full summaries of eccDNA structural annotations are shown in Table 1, and the distribution of eccDNA-encoded genes is summarized in a Venn diagram in Figure 3.

### 3.3. EccDNA Sequence Origins

To determine the genomic origins of eccDNA, a non-overlapping window approach was employed. Specifically, the genome was partitioned into consecutive 500 kbp windows, resulting in 4602 windows for assigning eccDNAs. The mean number of eccDNAs binned per window per condition was 2.50 ± 5.03, 1.97 ± 4.42, and 1.42 ± 3.89 for unadapted, 30-mM-adapted, and 60-mM-adapted, respectively. Windows in each condition with a Z-score ≥ 2 compared to the mean for each condition were considered statistically significant regions of high eccDNA biogenesis that may be considered hotspots. Approximately 1% of windows were considered significant across the three conditions with 48, 38, and 43 windows for unadapted, 30-mM-adapted, and 60-mM-adapted, respectively. The analysis of the genomic regions with the highest frequency of eccDNA biogenesis for each experimental condition revealed a notable representation of chromosome 9, spanning from 13 Mbp to 18 Mbp, with four windows ranging from 14 Mbp to 16 Mbp observed in all three conditions (Z ≥ 10). In the 60-mM-adapted sample, two windows exhibited higher biogenesis frequencies compared to the other samples. One of these windows was located in the telomeric region of chromosome 7, and the other was on chromosome X, containing nine and four genes, respectively, none of which were observed on eccDNAs. Although some variability was observed in other regions, the most prominent region of eccDNA formation remained unaffected by lactate adaptation. The top 15 windows with the highest frequency of eccDNA biogenesis for each condition are listed in Table 2. A comprehensive statistical analysis of eccDNA biogenesis mapping for all experimental conditions can be found in Supplemental Table S15, while genome-scale maps for each condition are presented in Supplemental Figures S1–S3.

### 3.4. Transcriptome Analysis

#### 3.4.1. EccDNA-Encoded Genes

It is plausible that genes encoded on eccDNAs may be transcriptionally active if the structures required for transcription are accurately replicated on the eccDNA from the template. To identify shifts that may be attributed to gene gain or loss, transcriptome data for genes encoded on eccDNAs were correlated. Out of 567 identified genes encoded on eccDNAs, 35 exhibited elevated levels of expression that were positively associated with the presence of an eccDNA-encoded copy of the gene. For the purpose of this analysis, an increase in transcript abundance was defined as a 1.2-fold change compared to the other conditions. Notably, these eccDNA-encoded genes were exclusively observed in one of the three experimental conditions.

**Figure 4:**
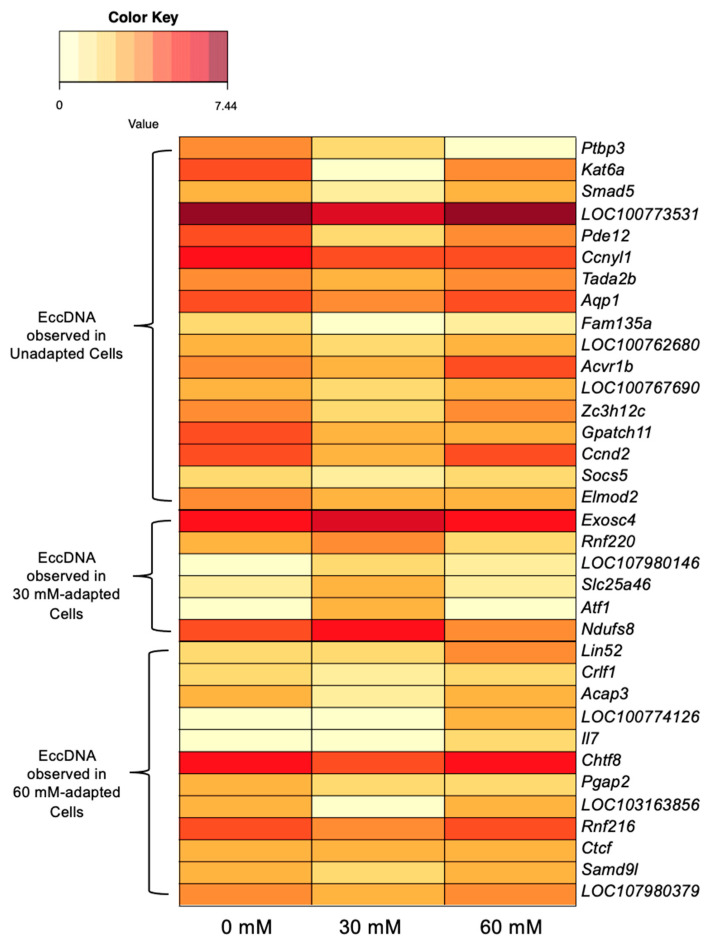
Heatmap of transcriptome abundance for eccDNA-encoded genes that show correlated expression with gene presence.

Out of the 567 identified genes encoded on eccDNAs, 17 were solely observed on eccDNAs in unadapted samples and displayed a reduced transcript abundance in 30-mM-adapted samples relative to unadapted samples. Among these genes were *Pde12* (Phosphodiesterase 12), which regulates mRNA stability in the mitochondrion [50] and functions as an exoribonuclease [51]; *Gpatch11* (G-Patch Domain Containing 11), a nucleic acid binding protein assumed to be present in kinetochores [52]; and *Ptbp3* (Polypyrimidine Tract Binding Protein 3), an RNA binding protein used as a biomarker in lung adenocarcinoma [53] and colorectal cancer [54]. In contrast, six eccDNA-encoded genes only observed in the 30-mM-adapted samples exhibited an increased transcript abundance in the 30-mM-adapted samples relative to the other conditions. These genes include *Ndufs8* (NADH:ubiquinone Oxidoreductase Core Subunit S8), which facilitates NADH oxidation and ubiquinone reduction in the electron transport chain [55], and *Exosc4* (Exosome Component 4), a component of exosomes that has been shown to stimulate cell proliferation and act as an oncogene [56]. In the 60-mM-adapted samples, 12 genes were exclusively detected on an eccDNA and displayed an elevated transcript abundance relative to the 30-mM-adapted samples. *Lin52* (Lin-52 DREAM MuvB Core Complex Component), which encodes a protein involved in DNA transcription and is part of the DREAM complex that inhibits cell cycle genes unless the oncogene *Mybl2* (MYB Proto-Oncogene Like 2) is overexpressed [57], was among the identified genes. Notably, the selectable marker for the VRC01 cell line, *Dhfr* (Dihydrofolate Reductase) [58], was observed on eccDNAs in unadapted and 30-mM-adapted samples, although the criteria for a 1.2-fold difference in transcript abundance was not met. The expression of *Dhfr* was comparable between these two conditions (TMM = 9.65 and 9.11, respectively) but decreased in the 60-mM-adapted cells, where it was not identified on an eccDNA (TMM = 7.76, 1.17-fold difference). Heatmaps of gene expression data that correlate with the presence of eccDNA-encoded genes can be found in Figure 4, and numerical data used to generate the figure as well as additional gene descriptions are located in Supplemental Table S16. Global gene expression values are provided in Supplemental Table S17.

#### 3.4.2. Metabolism-Linked Genes

**Figure 5:**
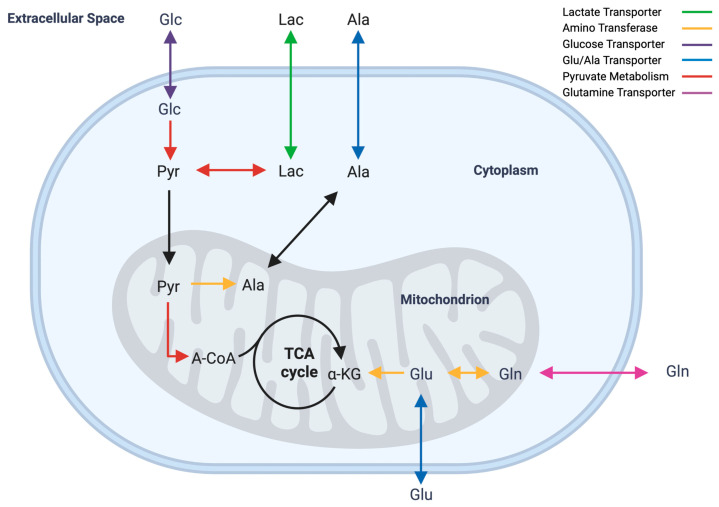
Simplified diagram of lactate and alanine metabolism. Arrows are representative of genes and gene families that facilitate reactions and transport. Green—monocarboxylate transporters, yellow—aminotransferases, purple—glucose transporters, blue—glutamate and alanine transporters, red—pyruvate-metabolism-associated genes, and pink—glutamine transporters. Black arrows reflect TCA cycle pathways and mitochondrial transport. Genes and gene families depicted in color were examined for differential expression (Figure 6). Figure generated using BioRender.

**Figure 6:**
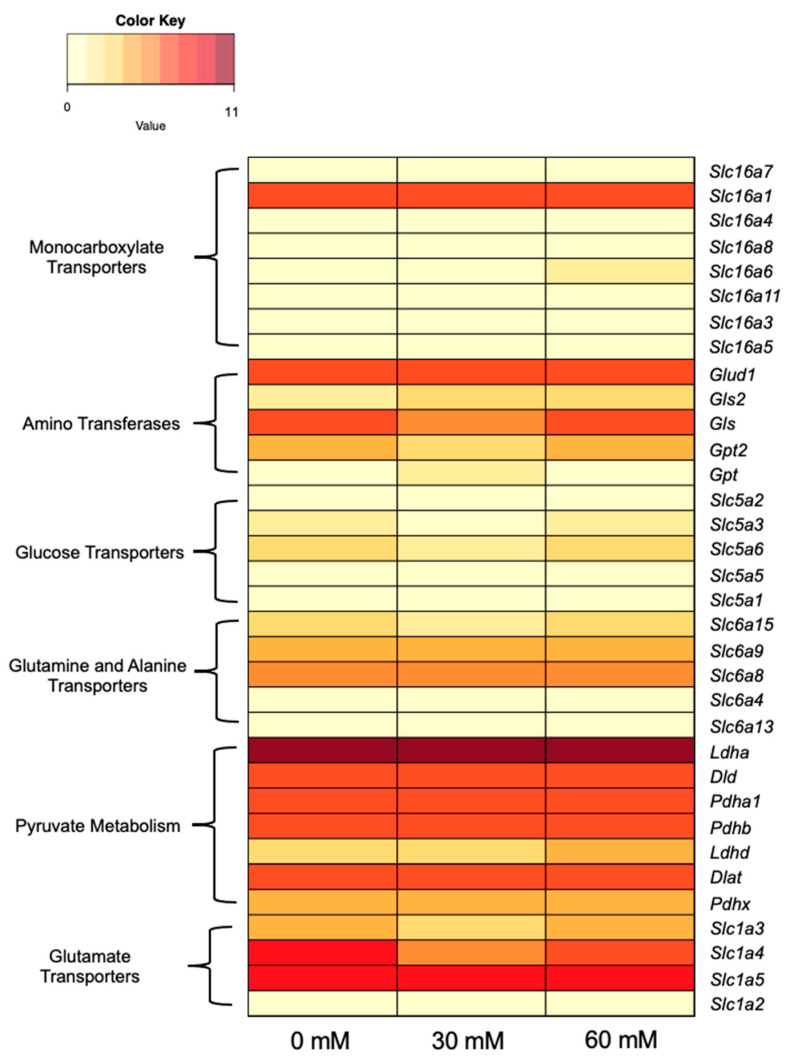
Heat map of RNA-seq data for genes facilitating lactate and alanine metabolism. Genes are grouped by function on the left y-axis and gene names are shown on the right y-axis. Lactate adaptation level is shown on the x-axis.

Although genes related to the lactate-adapted phenotype were not found on eccDNAs, the expression of genes involved in lactate and alanine metabolic pathways was investigated. These genes were categorized into six groups: monocarboxylate transporters (Slc16 family [59]), amino transferases [60], glucose transporters (Slc5 family [61]), alanine and glutamine transporters (Slc6 family [62]), pyruvate metabolism [60], and glutamate transporters (Slc1 family [63]). This simplified pathway is shown in Figure 5. Transcriptome data showed no substantial up- or downregulation in these genes in response to lactate adaptation aside from minor variation. *Slc16a6* and *Ldhd* showed slightly elevated levels of transcript abundance in 60-mM-adapted samples compared to unadapted samples; however, neither gene was determined to be differentially expressed with edgeR. A heatmap of RNA-seq data for lactate- and alanine-metabolism-associated genes is shown in Figure 6. Numerical data used to generate Figure 6 are available in Supplemental Table S18.

#### 3.4.3. Differentially Expressed Genes

Although the expression of genes related to lactate metabolism appears to remain consistent, significant alterations in gene expression (≤−2 or ≥2 log_2_ fold change) were detected throughout the adaptation process. Specifically, 30-mM-adapted cells exhibited 762 downregulated and 374 upregulated genes relative to unadapted cells. Comparison between 30-mM-adapted and 60-mM-adapted cells revealed 333 downregulated and 794 upregulated genes for 60-mM-adapted cells. Gene clustering using edgeR identified 1069 differentially expressed genes for 30-mM-adapted samples, with no net expression changes between unadapted and 60-mM-adapted samples. Among these, 868 genes were expressed at lower levels and 201 were expressed at higher levels in 30-mM-adapted samples. The analysis of the 1069 genes via KEGG pathway mapping revealed small numbers of genes involved in a broad range of functions across hundreds of pathways. Lists of genes upregulated and downregulated in 30-mM-adapted samples can be found in Supplemental Tables S19 and S20, respectively. Summaries of identified KEGG pathways for genes differentially expressed for 30-mM-adapted samples can be found in Supplemental Tables S21 and S22 and edgeR clusters are shown in Supplemental Figure S4.

Upon comparing unadapted cells to 60-mM-adapted cells, 134 genes were differentially expressed with 67 downregulated and 67 upregulated. Genes observed to have a net change in expression impacted many biological processes and may have a role in genome stability. *Rras* (RAS related), a GTPase that regulates angiogenesis and is often overexpressed in cancer [64], was found to have a 3.06-fold increase in expression levels. Another gene with a notable net overexpression was *Nox4* (NADPH oxidase 4), which catalyzes the production of reactive oxygen species (ROS) and plays an important role in hypoxia signaling. Further, *Nox4* allows for oncogenic adaptation in cancer by facilitating a metabolic shift that is less dependent on aerobic conditions [65]. A critical gene for DNA double-strand break repair, *Brca1* (BRCA1 DNA Repair Associated) [66], had a 2.71-fold decrease in expression by the end of lactate adaptation. The gene with the greatest net downregulation, *Hnrnpc* (Heterogeneous Nuclear Ribomucleoprotein C), is a regulator of pre-mRNA processing and has been observed to be overexpressed in some cancers; however, a reduced expression of *Hnrnpc* has been shown to limit tumor proliferation [67,68]. Genes with a net differential expression change are shown in Table 3. Descriptions of genes in Table 3 are shown in Supplemental Table S23.

## 4. Discussion

Controlling lactate and ammonia concentrations in industrial cell cultures is of great interest to the bioprocessing community as elevated concentrations can limit cell growth and lead to reduced productivity and product quality [1,4,49,69,70]. Numerous process engineering efforts have been made to mitigate waste production such as feeding alternative carbon sources [13,71], using sensors in controlled bioreactors to tune the feeding of glucose and/or glutamine [72,73], and varying culture pH [74,75]. Other studies have focused on choosing clones that have higher lactate consumption rates [20,76,77]. Genetic engineering attempts include the overexpression of pyruvate carboxylase 2 (*Pyc2*) [78,79], and galactokinase (*Galk1*) [6], as well as the knockdown or downregulation of lactate dehydrogenase A (*Ldha*) and/or pyruvate dehydrogenase kinases (*Pdhk*) [80,81,82,83]. While there is a large body of literature on controlling lactate levels and adapting cells to grow in lactate, results have been varied, with little consensus regarding the most efficient method. Further, the underlying mechanisms of these effects have not been deciphered.

Few studies have addressed the issue of feeding cultures with lactate to shift pH [5] or adapting cells to lactate with media supplementation [84]. Freund et al. authored the only published study characterizing a CHO cell line that is adapted to grow in elevated extracellular lactate. Notably, Freund et al. proposed that adaptation was likely a result of mass action rather than a specific metabolic shift that enabled adaptation. Notably, previous work in CHO cells has demonstrated that the amination of pyruvate to alanine is a metabolic stress response to excessive ammonia accumulation [23]. Phenotypic data of lactate-adapted cells in this work suggest that excessive lactate is likely mitigated in a similar fashion: by dehydrogenating lactate to produce pyruvate and transamination with α-ketoglutarate to form alanine. This hypothesis is supported by the reduced levels of ammonia observed in lactate-supplemented cultures of lactate-adapted cells (Figure 2G), which is consistent with the findings presented in Freund et al.’s lactate-adapted cells [84]. Alanine biosynthesis allows for the simultaneous removal of both lactate and ammonia (Figure 5D,G) and is likely a stress response mechanism for metabolic waste accumulation in CHO cells.

Overall, the eccDNA structure does not show significant signs of change with lactate adaptation (Table 1). Small shifts were observed, such as LINE and SINE elements increasing and decreasing; however, the shifts are so small they could easily be attributed to natural variation or variation from sample preparation and/or sequencing. The most substantial difference observed with respect to lactate adaptation was the increase in the average eccDNA sequence length combined with an increase in the proportion of eccDNAs harboring genes. An increase in the average sequence length could be indicative of recombination between smaller eccDNAs [27]; however, an in-depth, sequence-by-sequence structural analysis would be required to confirm this hypothesis. The proportions of repeat content and distribution, GC content, tRNA, and gene content exhibit consistency with minimal variation noted between lactate-adapted eccDNAs and the three conditions previously observed in VRC01 eccDNAs [49]. Collectively, these data suggest that the composition of eccDNA sequence structures in VRC01 is minimally impacted by stress adaptation and fed-batch culturing.

The identification of eccDNA biogenesis sites indicated a genomic hotspot on chromosome 9, specifically between 12.5 Mbp and 18 Mbp, in all levels of lactate adaptation (Table 2 and Supplemental Figures S1–S3). One genomic region on chromosome X exhibited a high biogenesis frequency only in the 60-mM-adapted samples, ranking among the top 15 regions with the highest frequencies. This region, which has been previously identified as a high-frequency window in other studies, was otherwise consistent in VRC01 [49]. Previous studies have demonstrated that eccDNA biogenesis frequencies can significantly shift in response to stress adaptation [27]; however, in this study, no significant changes in biogenesis frequencies were observed in response to lactate adaptation. The observed increase in frequencies from windows on chromosomes 7 and X in 60-mM-adapted eccDNAs cannot be attributed to transcriptome-driven biogenesis, as none of the genes located in these regions were found on an eccDNA. Notably, the chromosome 7 window with high frequencies is located at the telomeric region of the chromosome and may indicate a decreasing chromosome stability via the production of t-circles [85,86].

EccDNAs have been shown to mediate the stress response and adaptation via gene overexpression across species in response to many stimuli [27,31,87,88,89,90]. Further, some evidence has been shown that eccDNAs may play a minor role in modifying gene expression patterns in VRC01 cells grown in lactate-stressed conditions by amplifying expression of *Akr1b1* [49]. It is important to note that in the previous study, genes that correlated with an eccDNA gene presence had a more substantial difference in transcript abundance (≤−2 or ≥2 log_2_ fold change). Initially, employing the same threshold to this dataset showed no correlation, which resulted in the relaxed criteria utilized to identify correlation. Further, genes with a low level of expression (TMM < 2 in all three conditions) were excluded to limit false positives. Correlating transcript abundance with eccDNA gene content and establishing a 1.2× difference in TMM values identified 35 eccDNA-encoded genes that may be transcriptionally active.

RNA-seq data shown in this study suggest that certain changes in gene expression may be attributed to eccDNA; however, none of the eccDNA-encoded genes with correlated gene expression changes appear to respond to stress. In the 30-mM-adapted samples, *Ndufs8*, which is encoded on an eccDNA, is the only gene related to redox balancing that may have an impact on lactate adaptation, but this gene was not found on an eccDNA in the 60-mM-adapted samples. Two eccDNA-encoded genes that correlated with gene expression changes exhibit potential to enhance genome stability. Lin52 is a subunit of the DREAM complex, which is a crucial regulator of gene expression shifts that are dependent on cell cycle progression [91]. The overabundance of DREAM complex components could result in a tighter control of cell cycle genes that regulate growth and DNA synthesis. The downregulation of *Ptbp3* is also a positive sign for improving genome stability as overexpression is a highly reliable biomarker for cancers [53,54,92,93]. While the overexpression of *Ptbp3* is correlated with many cancer phenotypes, the knockdown or silencing of *Ptbp3* has been shown to induce cell cycle arrest and apoptosis [94].

Key metabolism and transporter genes were examined to identify shifts in gene expression that may accommodate the lactate-adapted phenotype. Despite the adaptation of cells to levels of extracellular lactate far beyond what is normally tolerable in CHO cell cultures, no evidence was found that indicates a transcriptome-level response. Freund et al. proposed that lactate adaption occurred by mass action; excess lactate in the culture would make conversion to pyruvate more thermodynamically favorable. While the lactate consumption phenotype is likely due to substrate-level control, lactate accumulated in all cultures until Day 3 regardless of lactate adaptation and/or supplementation, which would not agree with Freund’s proposed mechanism.

Significant changes in gene expression were observed between 60-mM-adapted and 30-mM-adapted samples, although lactate metabolism genes were not significantly modulated. In contrast, no such changes were observed between unadapted samples and 60-mM-adapted samples. Eukaryotic gene expression is regulated by complex signaling cascades [95]. Changes in expression are affected greatly by external signaling pathways and transcription factors, which can modulate expression for a short or long term [96]. The large shifts in gene expression with regard to 30-mM-adapted cells were unexpected; however, when comparing unadapted to 60-mM-adapted cells, some of the expression changes seen in 30-mM-adapted cells are likely attributed to gene expression tuning in response to the lactate stress [97]. Responding to a temporary stress can cause shifts in complex gene regulation pathways; adaptation to chronic stresses can cause the drastic rebalancing of regulatory cascades [96]. In yeasts, stochastic switching has been observed as a survival tactic in response to environmental stresses [98]. The observed random gene expression shifts in 30-mM-adapted cells could be attributed to the rapid pace of adaptation to lactate. Stochastic expression in response to stress has also been observed in Arabidopsis. For instance, when exposed to heat stress, more than 16,000 genes exhibited differential expression, with only 43 conferring a selective advantage for adaptation [99].

Examining net differential gene expression between unadapted and 60-mM-adapted samples identified 134 genes with a net change in transcript abundance by the end of the adaptation process. The increased expression of *Nox4* is likely the closest indicator of a gene expression shift mediating the lactate adaptation process as *Nox4* has been shown to encourage tumor growth in anaerobic environments [65]. Also notable is the downregulation of four splice variants of Brca1, a critical DNA repair gene. The maintenance of the *Brca1* gene is critical as excess stress can result in unintentional genomic rearrangements [100]. Further, the differential expression of *Rras* and *Hnrnpc* in opposite patterns is also unusual, as the overexpression of these genes typically encourages proliferation. The data presented in this study provide a single point in time snapshot of a highly dynamic lactate adaptation process, and although gene expression may become more finely tuned at higher levels of adaptation, it is probable that stochastic switching may still play a role. To identify precise changes in gene expression in response to lactate adaptation, time-series data from extended cultures of lactate-adapted cells would be necessary.

## 5. Conclusions

This study characterizes phenotypic behavior, circulome dynamics, and gene expression shifts in CHO cells gradually adapted to grow in extreme levels of extracellular lactate. The generation of alanine in lactate-adapted cultures that are supplemented with lactate suggests that alanine biosynthesis may be an efficient mitigation mechanism of both lactate and ammonia in CHO cells. EccDNA content in the VRC01 cell line remains highly heterogeneous and dynamic with no clear evidence of contributing to overall differential gene expression in a substantial way. Notably, eccDNAs were observed to be longer and less abundant as lactate adaptation progressed, which may be indicative of eccDNA recombination. The underlying mechanism of lactate adaptation in CHO cells remains unclear, though the expression of lactate metabolism genes does not appear to change in response to lactate stress. Finally, 134 genes were found to have a net change in expression through the adaptation process, though more than 1000 genes were observed to be differentially expressed between unadapted, 30-mM-adapted, and 60-mM-adapted cultures. These data could suggest the stochastic switching of gene expression as a stress adaptation mechanism in CHO cells. The further study of these gene expression profiles in these cells grown in an extended culture is needed to fully assess the contribution to the lactate-adapted phenotype.

## Figures and Tables

**Table 1:** Sequence characteristics of eccDNA sequences observed in CHO cells at varying levels of lactate adaptation. Repeat motif content, gene content, tRNA content, rDNA content, and potential origins of replications are included. Sequences were pooled by lactate adaptation level and clustered for sequence similarity (>90%).

Condition	Sequences	Sequences Clustered	Max Length (bp)	Average Length (bp)	Repeat bases masked	GC (%)	eccDNA with genes	eccDNA with rDNA	eccDNA with tRNA	ORI (>95%)
**Unadapted**	8202	8109	24,436	4905	15,159,445 bp (37.9%)	40.6%	295 (3.6%)	6	800 (9.9%)	3
**30 mM Adapted**	6476	6419	27,326	4976	12,132,410 bp (37.9%)	40.5%	255 (4.0%)	4	618 (9.6%)	3
**60 mM Adapted**	4563	4511	24,598	5515	9,671,540 bp (38.9%)	40.3%	233 (5.2%)	4	484 (10.7%)	4
		**Unadapted**			**30 mM Adapted**			**60 mM Adapted**		
**Repeat Structure**	**Subcategory**	**Number of Sequences**	**Base pairs (bp)**	**Percent of total bases**	**Number of Sequences**	**Base pairs (bp)**	**Percent of total bases**	**Number of Sequences**	**Base pairs (bp)**	**Percent of total bases**
**SINEs:**		23,088	2,994,270	7.49	17,928	2,322,980	7.25	13,529	1,750,013	7.03
	**Alu/B1**	9915	1,172,210	2.93	7715	910,743	2.84	6008	712,601	2.86
	**MIRs**	1263	142,664	0.36	972	108,753	0.34	688	77,202	0.31
**LINEs:**		12,326	5,818,281	14.6	9816	4,729,537	14.8	7735	3,856,083	15.5
	**LINE1**	11,416	5,678,568	14.2	9140	4,623,778	14.4	7284	3,781,556	15.2
	**LINE2**	725	111,589	0.28	564	88,862	0.28	359	62,787	0.25
	**L3/CR1**	136	20,991	0.05	81	11,098	0.03	68	8645	0.03
	**RTE**	45	6525	0.02	26	5024	0.02	22	2683	0.01
**LTR elements:**		13,196	3,972,926	9.94	10,381	3,175,847	9.91	8222	2,552,906	10.3
	**ERVL**	1223	349,001	0.87	888	267,321	0.83	795	217,908	0.88
	**ERVL-MaLRs**	5550	1,498,301	3.75	4444	1,207,001	3.77	3434	927,879	3.73
	**ERR_class I**	1259	262,964	0.66	972	189,106	0.59	799	172,688	0.69
	**ERV_class II**	5043	1,804,631	4.52	3991	1,464,909	4.57	3115	1,182,229	4.75
**DNA elements:**		2334	431,383	1.08	1899	358,406	1.12	1363	250,375	1.01
	**hAT-Charlie**	1415	247,910	0.62	1173	205,506	0.64	832	147,135	0.59
	**TcMar-Tigger**	598	123,340	0.31	456	98,914	0.31	326	69,009	0.28
**Unclassified:**		626	244,548	0.61	464	197,627	0.62	389	141,496	0.57
**Total Interspaced Repeats:**		-	13,461,408	33.7	-	10,784,397	33.7	-	8,550,873	34.4
**Small RNA:**		581	41,990	0.11	467	34,506	0.11	412	31,344	0.13
**Satellites**		1694	727,205	1.82	1338	579,860	1.81	1112	491,622	1.98
**Simple Repeats**		16,679	836,271	2.09	13,098	665,925	2.08	10,325	541,927	2.18
**Low Complexity**		2008	105,297	0.26	1514	77,218	0.24	1231	62,244	0.25

**Table 2:** The 15 highest frequency eccDNA biogenesis windows observed for each condition.

**Unadapted**						
**Chr**	**Start**	**End**	**Binned EccDNAs**	**Binned EccDNAs with Genes (#)**	**Binned EccDNAs with Genes (%)**	**Z-score**
9	15,000,000	15,500,000	217	10	4.6	42.66
9	14,500,000	15,000,000	113	22	19.5	21.98
9	14,000,000	14,500,000	89	7	7.9	17.2
9	15,500,000	16,000,000	88	3	3.4	17
9	13,500,000	14,000,000	84	0	0	16.21
9	13,000,000	13,500,000	51	1	2	9.64
10	25,500,000	26,000,000	47	0	0	8.85
9	16,500,000	17,000,000	36	1	2.8	6.66
9	16,000,000	16,500,000	35	2	5.7	6.46
9	17,500,000	18,000,000	35	0	0	6.46
9	25,500,000	26,000,000	32	2	6.3	5.87
4	131,500,000	132,000,000	29	0	0	5.27
7	134,000,000	134,359,064	27	0	0	4.87
9	17,000,000	17,500,000	27	2	7.4	4.87
1_2	195,000,00	195,500,000	23	0	0	4.08
**30 mM-adapted**						
**Chr**	**Start**	**End**	**Binned EccDNAs**	**Binned EccDNAs with Genes (#)**	**Binned EccDNAs with Genes (%)**	**Z-score**
9	15,000,000	15,500,000	185	8	4.3	41.41
9	14,500,000	15,000,000	117	19	16.2	26.03
9	14,000,000	14,500,000	84	3	3.6	18.56
9	13,500,000	14,000,000	76	3	3.9	16.75
9	15,500,000	16,000,000	61	0	0	13.36
9	16,000,000	16,500,000	55	6	10.9	12
9	12,500,000	13,000,000	47	1	2.1	10.19
9	17,500,000	18,000,000	41	4	9.8	8.83
9	13,000,000	13,500,000	36	3	8.3	7.7
1_2	196,000,000	196,500,000	34	1	2.9	7.25
9	16,500,000	17,000,000	29	2	6.9	6.12
2	34,500,000	35,000,000	21	4	19	4.31
4	72,500,000	73,000,000	20	0	0	4.08
7	134,000,000	134,359,064	19	0	0	3.86
9	25,500,000	26,000,000	19	3	15.8	3.86
**60 mM-adapted**						
**Chr**	**Start**	**End**	**Binned EccDNAs**	**Binned EccDNAs with Genes (#)**	**Binned EccDNAs with Genes (%)**	**Z-score**
9	15,000,000	15,500,000	141	7	5	35.89
9	14,500,000	15,000,000	98	23	23.5	24.84
9	14,000,000	14,500,000	85	5	5.9	21.49
9	13,500,000	14,000,000	77	1	1.3	19.44
9	15,500,000	16,000,000	53	0	0	13.26
7	134,000,000	134,359,064	41	0	0	10.18
X	106,500,000	107,000,000	38	0	0	9.41
9	12,500,000	13,000,000	35	2	5.7	8.64
9	16,000,000	16,500,000	33	10	30.3	8.12
2	41,000,000	41,500,000	32	0	0	7.86
7	88,000,000	88,500,000	32	2	6.3	7.86
9	17,500,000	18,000,000	32	2	6.3	7.86
2	34,500,000	35,000,000	29	9	31	7.09
4	5,000,000	5,500,000	29	0	0	7.09
9	17,000,000	17,500,000	28	1	3.6	6.83

**Table 3:** Genes with significant shifts in net expression between unadapted and 60-mM-adapted CHO cells. Log2FC—log_2_ fold change is normalized to the unadapted cells.

RefSeq ID	Gene	log2FC	RefSeq ID	Gene	log2FC	RefSeq ID	Gene	log2FC
XP_027259257.1	*Pnisr*	5.08	XP_027263110.1	*Mms19*	2.3	XP_027262108.1	*C3H16orf87*	−2.43
XP_027271342.1	*LOC107977511*	4.72	XP_027268884.1	*Yeats2*	2.29	XP_035302467.1	*Asip*	−2.43
XP_035300595.1	*Camk2n2*	4.07	XP_027266487.1	*Banp*	2.28	XP_027250346.1	*Oard1*	−2.44
XP_027260962.1	*Usp6nl*	4.01	XP_027272584.1	*Trip10*	2.26	XP_035296751.1	*Prkag1*	−2.45
XP_027243872.1	*Spred2*	3.97	XP_027260324.1	*Kat8*	2.26	XP_027246344.1	*Tinf2*	−2.45
XP_035299495.1	*Vps11*	3.81	XP_027281260.1	*Baiap2*	2.25	XP_035309594.1	*LOC118237753*	−2.54
XP_027270425.1	*Ccdc51*	3.77	XP_027281261.1	*Baiap2*	2.25	XP_035296374.1	*Tmem161b*	−2.62
XP_027265866.1	*Tmem41b*	3.64	XP_027269839.1	*LOC100765617*	2.2	XP_027243712.1	*Mpv17l*	−2.62
XP_035309292.1	*Tdrd3*	3.52	XP_027279239.1	*Zswim3*	2.19	XP_027243713.1	*Mpv17l*	−2.62
XP_027284541.1	*Spr*	3.51	XP_027256563.1	*Spata24*	2.18	XP_035308661.1	*Mpv17l*	−2.62
XP_027274795.1	*Nit1*	3.33	XP_027253398.1	*Asb1*	2.18	XP_027285208.1	*Ino80b*	−2.7
XP_035301857.1	*Nit1*	3.33	XP_035301484.1	*Rbbp5*	2.18	XP_027285383.1	*Tra2a*	−2.71
XP_027258717.1	*Ccn2*	3.32	XP_027264466.1	*LOC100757535*	2.15	XP_027283850.1	*Brca1*	−2.71
XP_027278256.1	*Pex16*	3.16	XP_027277209.2	*Asip*	2.14	XP_027283851.1	*Brca1*	−2.71
XP_027282741.1	*CUNH17orf49*	3.09	XP_035296265.1	*Nhsl1*	2.08	XP_027283852.1	*Brca1*	−2.71
XP_027276511.1	*Rras*	3.06	XP_027258948.1	*Anp32b*	2.06	XP_027283853.1	*Brca1*	−2.71
XP_027268403.1	*Slc35a5*	3.05	XP_027254583.1	*Il7*	2.06	XP_027256348.1	*Rnf138*	−2.71
XP_027263390.1	*Nox4*	3.05	XP_027264703.1	*Mob2*	2.05	XP_027274946.1	*Atxn3*	−2.75
XP_027279804.1	*Coro7*	2.98	XP_027273144.1	*Acbd6*	2.04	XP_035298824.1	*Hivep1*	−2.77
XP_027254088.1	*H6pd*	2.95	XP_027281267.1	*Chmp6*	2.03	XP_027243022.1	*Ctbp1*	−2.82
XP_027268965.1	*Klhl22*	2.95	XP_027266778.1	*Mthfs*	2	XP_035298483.1	*Cpne2*	−2.83
XP_027247336.1	*Agk*	2.94	XP_027266779.1	*Mthfs*	2	XP_027245634.1	*Fam133b*	−2.87
XP_027246345.1	*Tinf2*	2.92	XP_035307155.1	*LOC100774954*	−2.04	XP_027271986.1	*Hip1*	−2.89
XP_027286836.2	*Zc3h4*	2.9	XP_027274041.1	*Trmt5*	−2.05	XP_027252162.1	*Pphln1*	−3.06
XP_027252267.1	*Cacnb3*	2.88	XP_027250449.1	*Nr2c2ap*	−2.07	XP_027287883.1	*Mospd1*	−3.11
XP_035306485.1	*Ptpn12*	2.87	XP_027258438.1	*Poli*	−2.08	XP_027244120.1	*Dnajb12*	−3.13
XP_027278991.1	*Ptpa*	2.86	XP_027274796.1	*Nit1*	−2.13	XP_027244121.1	*Dnajb12*	−3.13
XP_027259074.1	*Stoml2*	2.84	XP_027274797.1	*Nit1*	−2.13	XP_035304186.1	*Top3a*	−3.2
XP_027255528.1	*Id3*	2.83	XP_027274798.1	*Nit1*	−2.13	XP_027271702.1	*Pus1*	−3.2
XP_027275696.1	*Slco4a1*	2.81	XP_027274799.1	*Nit1*	−2.13	XP_027247306.1	*Hipk2*	−3.28
XP_027243830.1	*Commd1*	2.73	XP_027274800.1	*Nit1*	−2.13	XP_027258058.1	*Rtn4ip1*	−3.41
XP_035295692.1	*Ift74*	2.68	XP_027274801.1	*Nit1*	−2.13	XP_027262953.1	*Ldb1*	−3.47
XP_027252455.1	*Eif4b*	2.6	XP_027258090.1	*Pde4b*	−2.15	XP_027250779.1	*LOC103162709*	−3.66
XP_027288617.1	*Mospd2*	2.57	XP_027266034.1	*Eef2k*	−2.16	XP_027255726.1	*Zbtb17*	−3.67
XP_027265269.1	*Zkscan8*	2.56	XP_027288241.1	*Fam3a*	−2.18	XP_027270426.1	*Ccdc51*	−3.73
XP_035294787.1	*LOC113833870*	2.55	XP_035295624.1	*Ndc1*	−2.19	XP_027285126.1	*Atp6v1e1*	−3.87
XP_027282857.1	*Vamp2*	2.55	XP_035307073.1	*LOC100755006*	−2.21	XP_035303760.1	*Vamp2*	−3.91
XP_027283079.1	*Top3a*	2.46	XP_027267297.1	*Pde4a*	−2.21	XP_027266816.1	*Ube3d*	−3.95
XP_027283080.1	*Top3a*	2.46	XP_027288197.1	*Taz*	−2.23	XP_035298723.1	*Usp6nl*	−4.16
XP_027258844.1	*Palm2akap2*	2.42	XP_027265636.1	*Uimc1*	−2.27	XP_027266818.1	*Ube3d*	−4.33
XP_027271581.1	*Pnpla6*	2.4	XP_027269840.1	*LOC100765617*	−2.3	XP_027262827.1	*Smndc1*	−4.34
XP_027255969.1	*Exoc3*	2.4	XP_027248483.1	*Baz2a*	−2.32	XP_027250669.1	*Smim7*	−4.38
XP_027278905.1	*Rapgef1*	2.39	XP_027248484.1	*Baz2a*	−2.32	XP_027257570.1	*Tmem167a*	−5.29
XP_027248513.1	*Ankrd52*	2.34	XP_035306520.1	*Baz2a*	−2.32	XP_027242456.1	*Hnrnpc*	−5.62
XP_027257344.1	*Ppwd1*	2.34	XP_035300748.1	*Khsrp*	−2.38			

## Data Availability

All sequence data generated and/or analyzed in this study are available in the NCBI sequence read archive under BioProject: PRJNA992436 (Submission ID: SUB13644941). All other data generated or analyzed during this study are included in the published article and its supplementary information files.

## Supplementary Materials

The following supporting information can be downloaded at: https://www.mdpi.com/article/10.3390/genes14081576/s1, Figure S1: Unadapted eccDNA biogenesis map; Figure S2: 30-mM-adapted eccDNA biogenesis map; Figure S3: 60-mM-adapted eccDNA biogenesis map; Figure S4: edgeR clusters of genes differentially expressed in 30-mM-adapted samples relative to other conditions; Table S1: Origins of Replication Database; Table S2: rDNA Database; Table S3: Unadapted tRNA; Table S4: 30-mM-adapted tRNA; Table S5: 60-mM-adapted tRNA; Table S6: Unadapted ORI; Table S7: 30-mM-adapted ORI; Table S8: 60-mM-adapted ORI; Table S9: Unadapted rDNA; Table S10: 30-mM-adapted rDNA; Table S11: 60-mM-adapted rDNA; Table S12: Unadapted Maker output; Table S13: 30-mM-adapted Maker output; Table S14: 60-mM-adapted Maker output; Table S15: Biogenesis frequencies; Table S16: TMM values for Figure 5. Table S17: Global gene expression data; Table S18: TMM values for Figure 5 and Figure 6; Table S19: Genes upregulated in 30-mM-adapted samples relative to other conditions; Table S20: Genes downregulated in 60-mM-adapted samples relative to other conditions; Table S21: KEGG pathways of genes upregulated in 30-mM-adapted samples relative to other conditions; Table S22: KEGG pathways of genes downregulated in 30-mM-adapted samples relative to other conditions. Table S23: Descriptions of genes with significant shifts in net expression between unadapted and 60 mM-adapted CHO cells.
