# Supplementary figures and images for: Dynamics of Amino Acid Metabolism, Gene Expression, and Circulomics in a Recombinant Chinese Hamster Ovary Cell Line Adapted to Moderate and High Levels of Extracellular Lactate
Source: Genes (Basel). 2023 Aug 2;14(8):1576. doi: 10.3390/genes14081576 (PMC10454118; doi:10.3390/genes14081576)

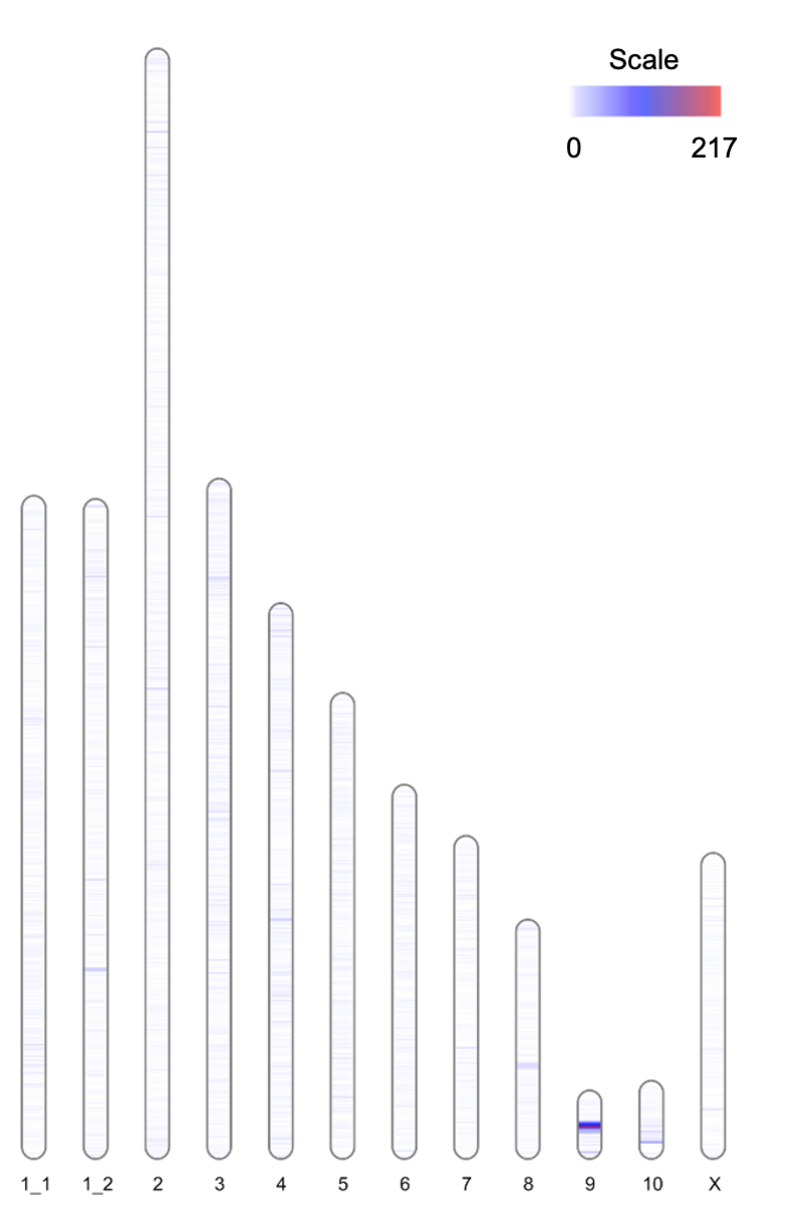

Supplement: Supplementary file 1 [file genes-14-01576-s001.zip › genes-2525718-supplementary/Genes_Supplemental_Info_v2/figs1redo.tif]

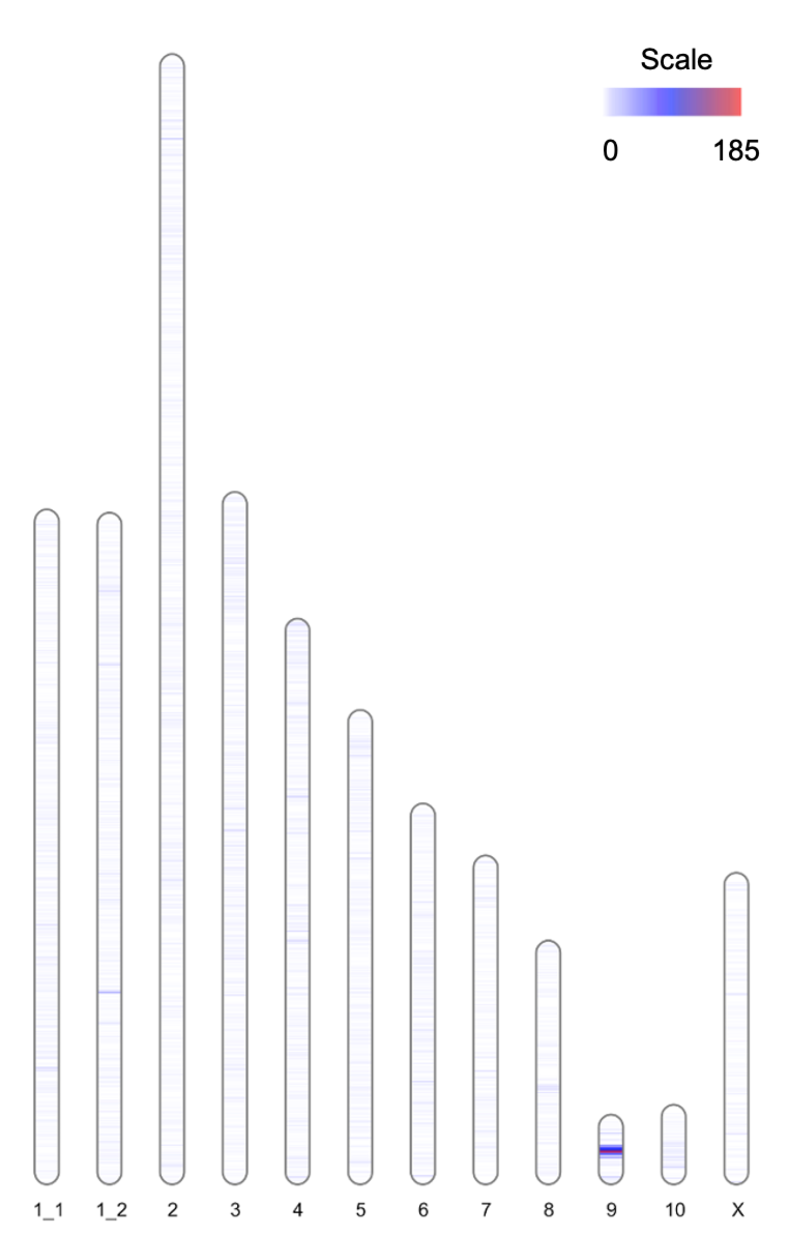

Supplement: Supplementary file 1 [file genes-14-01576-s001.zip › genes-2525718-supplementary/Genes_Supplemental_Info_v2/figs2redo.tif]

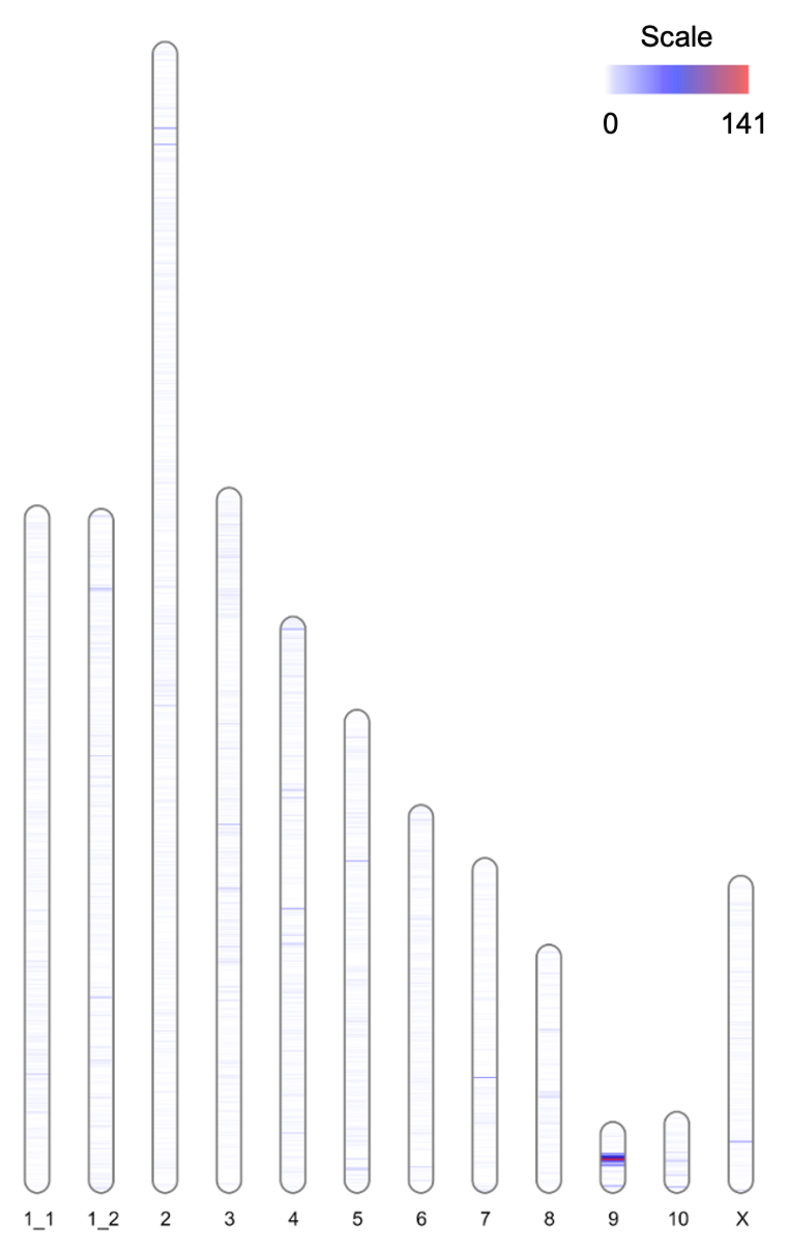

Supplement: Supplementary file 1 [file genes-14-01576-s001.zip › genes-2525718-supplementary/Genes_Supplemental_Info_v2/figs3redo.tif]

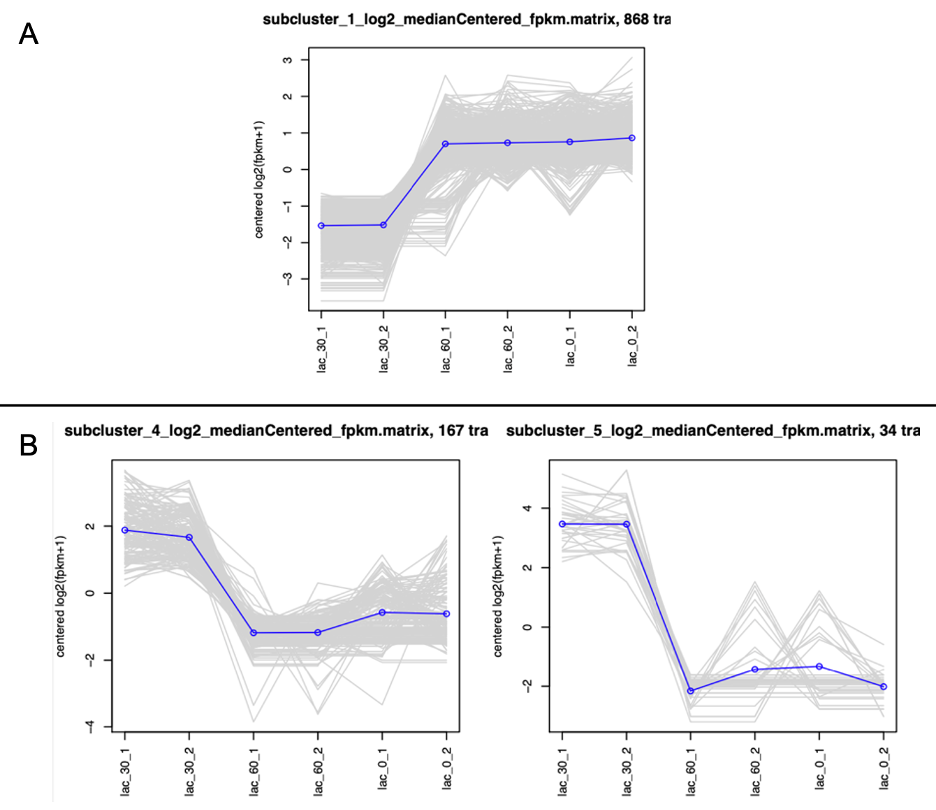

Supplement: Supplementary file 1 [file genes-14-01576-s001.zip › genes-2525718-supplementary/Genes_Supplemental_Info_v2/figS4.tif]
